# Squamous Cell Carcinoma of the Esophagus and Adenocarcinoma of Gastroesophageal Junction, a Rare Coincidence

**Published:** 2011-07-01

**Authors:** B Geramizadeh, A Safari, F Javadi, Sh Bolandparvaz

**Affiliations:** 1 Transplant Research Center, Shiraz University of Medical Sciences, Shiraz, Iran; 2 Department of Pathology, Shiraz University of Medical Sciences, Shiraz, Iran; 3 Trauma Research Center, Shiraz University of Medical Sciences, Shiraz, Iran; 4 Department of Surgery, Shiraz University of Medical Sciences, Shiraz, Iran

**Keywords:** Stomach, Esophagus, Squamous cell carcinoma, Adenocarcinoma

## Abstract

Simultaneous squamous cell carcinoma (SCC) of esophagus and gastroesophageal adenocarcinoma has rarely been reported. It is often difficult to diagnose this coexistence preoperatively due to the presence of esophageal stenosis. Herein, we report a patient with esophageal SCC whose gastroesophageal adenocarcinoma was also detected after pathologic examination of the resected specimen

## Introduction

The simultaneous presence of esophageal squamous cell carcinoma (SCC) and gastric adenocarcinoma is very uncommon. There are rare case reports in the English literature.[1]

In this report, we present our first experience (during 20 years) of an 80-year-old man with esophageal SCC in which gastroesophageal adenocarcinoma was incidentally detected after surgery in the margin of surgical resection.

## Case Report

An 80-year-old man presented with progressive dysphagia, weakness and weight loss since 5 months prior to admission. His past medical history was completely unremarkable. Physical examination showed a cachectic and pale old male. Heart, lung and other parts of the body were unremarkable. Laboratory findings were: WBC: 3400/ml, Hb: 7.3 gr/dl, platelet: 190000/ml. Occult blood was detected in the stool examination. Upper gastrointestinal endoscopy showed an ulcerative infiltrative mass in the lower third of esophagus. The lumen of the esophagus was narrowed by the tumor so that the tube could not be passed through stomach. A biopsy was taken with the pathologic diagnosis of well differentiated SCC.

CT scan of the chest and abdomen did not reveal any additional finding without any evidence of metastasis. The patient underwent surgery for resection of the tumor, and distal esophagectomy (Orringer's operation) was performed. The specimen received in the pathology laboratory consisted of a segment of esophagus with an ulcerated infiltrative mass measuring 5Xx3x2.5 cm which has nearly obliterated the lumen with 2-cm distance from the distal resected margin of cardia which grossly seemed unremarkable except for mild firmness. Grossly, no overt mass was detected in the stomach.

Microscopic analysis of the sections from the esophagus showed well differentiated SCC invading the adventitia (Figure 1A). The distal surgical resection margin of gastroesophageal junction showed well differentiated adenocarcinoma with focal invasion to the superficial muscularis properia (Figure 1B). There was normal esophageal mucosa between the two tumors, so these two tumors were completely separate. No lymph node metastasis of either SCC or adenocarcinoma was identified.

**Fig. 1A:**
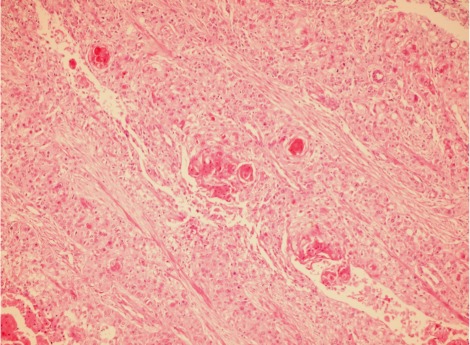
Sections from esophageal SCC

**Fig. 1B:**
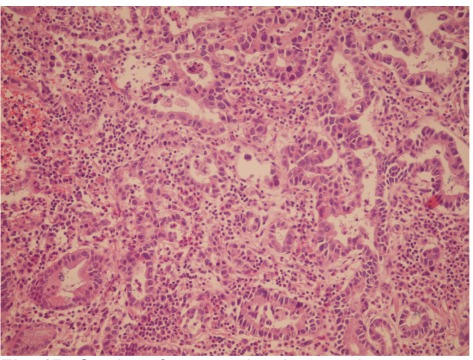
Dections from gastric adenocarcinoma

Unfortunately after resection of the esophagus, the patient developed pneumothorax and with the diagnosis of air leak and right main bronchus rupture, emergency right postrolateral thoracotomy performed and chest tube was inserted but the patient did not tolerate and died after cardiorespiratory arrest.

## Discussion

Esophageal cancer is the 8th most common cancer and the sixth most common cause of death from cancer worldwide.2-4 SCC is the predominant histologic picture, but its coincidence with gastroesophageal adenocarcinoma has rarely been reported.5 Synchronous cancer was defined as a primary carcinoma of the extraesophageal organs detected within 1 year of the diagnosis of esophageal carcinoma based on the criteria reported by Warren and Gates.[6]

In a report from Japan, with a high incidence of both esophageal SCC and gastric adenocarcinoma, just 2.1% of 11732 patients with esophageal SCC, had synchronous cancers of other organs, the most common of which had been gastric early adenocarcinoma,[7][8] but in Western countries, the most common synchronous cancer with esophageal SCC were from oral cavity, larynx and pharynx.[9] However, incidental finding of adenocarcinoma of gastroesophageal junction during surgery of esophageal SCC has very rare-ly been reported.[1][10]

All the previously reported cases have been in old male patients (>60 years of age). Diet, cigarette smoking and alcohol consumption have been considered as the main risk factors of this coincidence.[3] Our patient has been a heavy cigarette smoker for more than 50 years but without any history of alcohol consumption. The most common presentation in previous cases was dysphagia and abdominal discomfort, such as our patient.[3]The main therapeutic strategy in these cases was surgical exploration and excision of both tumors with postoperative adjuvant chemoradiation,[11] which was not possible in our patient because of the lack of diagnosis of the second cancer before surgery and the patient's death shortly after surgery.Based on our experience in this report, we want to stress on the importance of detailed preoperative gastric examination and of careful intraoperative inspection of gastric mucosa in patients with esophageal cancer whose preoperative gastric examination pro-vides inconclusive results due to the presence of severe esophageal stenosis.

## References

[1] Rodriges Santos CE, Accetta AC, Riello de Mello EL, Martins de Oliveira I, de Freitas Machado LF, de Almeida Dias J (2006). Collision tumor at the esophagogastric junction: a case report. Applied Cancer Research.

[2] Ziaian B, Montazeri V, Khazaiee R, Amini S, Karimi M, Mehrabani D (2010). Esophageal cancer occurrence in Southeastern Iran.. J Res Med Sci.

[3] Mehrabani D, Tabei SZ, Heydari ST, Shamsina SJ, Shokrpour N, Amini M, Masoumi SJ, Julaee H, Farah mand M, Manafi A (2008). Cancer occurrence in Fars Province, Southern Iran.. Iran Red Crescent Med J.

[4] deh D, Møller H, Boffetta P, Malekzadeh R (2009). Oesophageal cancer in Golestan province, a high incidence area in northern Iran- A review.. European J Cancer.

[5] Reider R (2003). A rare case of simultaneous gastric adenocarcinoma and squamous cell carcinoma of the esophagus in a Pruvian, geriatric, female. Dig Dis Sci.

[6] Lim SK, Sampson CC, Warner OG (1981). Simultaneous primary carcinomas-- a report of three cases. J Natl Med Assoc.

[7] Suzuki S, Nishimaki T, Suzuki T, Kanda T, Nakagawa S, Hatakeyama K (2002). Outcomes of simultaneous resec-tion of synchronous esophageal and extraesophageal carcinomas. J Am Coll Surg.

[8] Nakayama K, Abo S (1979). Concurrent cancer of the esophagus in Japan. Int Adv Surg Oncol.

[9] Chuang SC, Hashibe M, Scelo G, Brewster DH, Pukkala E, Friis S, Tracey E, Weiderpass E, Hemminki K, Tamaro S, Chia KS, Pompe-Kirn V, Kliewer EV, Tonita JM, Martos C, Jonasson JG, Dresler CM, Boffetta P, Brennan P (2008). Risk of second primary cancer among esophageal cancer patients: a pooled analysis of 13 cancer registeries. Cancer Epidemiol Biomarkers Prev.

[10] Souquet JC, Berger F, Bonvoisin S, Partensky C, Boulez J, Descos F, Lambert R (1989). Esophageal squamous cell carcinoma associated with gastric adenocarcinoma. Cancer.

[11] Maeta M, Koga S, Kimura A (1991). Simultaneous superficial squamous cell carcinoma of the esophagus and early gastric adenocarcinoma. Hep-atogastroenterology.

